# Development and Upgrade of a Robot That Enables Three‐Dimensional Trunk Motion and Lower Limb Exercise for Stroke Patients: A Pilot Clinical Investigation Including a User Feasibility Test

**DOI:** 10.1155/bmri/5515943

**Published:** 2026-08-05

**Authors:** Justin Kim, Philbert Chen, Jacob Kim, Heegoo Kim, Phillip Siwoo Kim, MinYoung Kim

**Affiliations:** ^1^ Carle Illinois College of Medicine, University of Illinois at Urbana-Champaign, Urbana, Illinois, USA, illinois.edu; ^2^ Department of Occupational & Environmental Medicine, Carle Illinois College of Medicine University of Illinois at Urbana-Champaign, Urbana, Illinois, USA; ^3^ University of Pennsylvania, Philadelphia, Pennsylvania, USA, upenn.edu; ^4^ Department of Rehabilitation Medicine, CHA Bundang Medical Center, CHA University School of Medicine, Seongnam, Republic of Korea, cha.ac.kr; ^5^ Digital Therapeutics Research Team, CHA Bundang Medical Center, CHA University School of Medicine, Seongnam, Republic of Korea, cha.ac.kr; ^6^ School of Arts and Science, Case Western Reserve University, Cleveland, Ohio, USA, case.edu

**Keywords:** gait training, robot, satisfaction, stroke, trunk motion

## Abstract

**Background:**

Stroke impairs functional independence including gait ability. While robot‐aided rehabilitation techniques have been developed, there is a need for a robotic system that promotes three‐dimensional (3D) movement of the trunk and gait ability while providing automatic function evaluation and individually optimized prescriptions for stroke patients.

**Objective:**

To assess the efficacy and safety of a novel trunk and lower limb robotic system for patients with hemiplegia due to chronic stroke.

**Methods:**

This study employed a single‐arm, before‐after trial design conducted in an institutional setting. Twenty individuals (eight males; mean age, 52.3 ± 11.8 years) with hemiplegia resulting from chronic stroke (≥ 12 months postonset) who showed no change in the Functional Ambulation Category (FAC) for at least 2 months prior to baseline were enrolled. Participants underwent robot‐assisted training using a system composed of trunk, upright, and lower extremity modules. Each participant received 30‐min sessions, 5 days per week, for 4 weeks. The robotic system provided real‐time kinematic motion data to support rehabilitation exercises. Primary outcomes included changes in functional evaluation and performance scores from baseline to postintervention. Additionally, a feasibility survey was administered to assess user satisfaction and identify areas for system improvement.

**Results:**

In all patients, the functional scores did not differ significantly between baseline and 1 month after the robotic intervention. However, there were improvements in the performance scores of lateral and forward bending of the trunk while sitting, forward bending ability during sit‐to‐stand activity, weight transfer, and lateral and forward bending of the trunk while standing 1 month after the intervention (*p* < 0.05). The user experience survey showed satisfaction in 16 of 20 patients due to the easy and safe operation of the robot.

**Conclusions:**

The proposed robotic system was safe and convenient to use and was associated with improvements in selected kinematic parameters.

## 1. Introduction

Stroke is one of the most common causes of major disabilities worldwide [1]. Of survivors who live at least 5 years after the stroke event, approximately 30%–50% achieve suboptimal neurophysiological functioning through measures of motor ability, cognition, and activities of daily living [2]. Among these neurological deficits, obvious hemiplegia is a frequent condition and poses a severe burden on patients and their families [3]. So far, traditional physical and occupational therapy is the primary treatment option for hemiplegia due to stroke; however, this is labor‐intensive, and its effect might depend on the therapist′s ability and competence [4]. Furthermore, evaluations of the patients′ functional ability from the initial stage after stroke to the follow‐up stage after rehabilitation therapy are often subjective in the clinic.

In recent years, robot‐aided neurorehabilitation techniques have been extensively developed due to the increased sophistication of electromechanical engineering and new computational approaches [5–9]. Previous studies reported greater neurological and functional improvements by robotic sensorimotor training, which were concomitantly provided with traditional rehabilitation, compared to only traditional rehabilitation in stroke patients [10, 11]. Technological advancement in relevant engineering has made robotics available for rehabilitation interventions [12].

To achieve normal gait patterns, balance reactions, including postural tone and sensation, should be actively engaged in a gait sequence with minimal effort. Most stroke patients struggle with selective control of the trunk and lower extremities, leading to difficulties in postural control and gait [13]. Thus, regaining trunk control and balance is a significant element in stroke rehabilitation to enhance the ability to walk [14–17]. However, most gait training robots currently on the market or under development focus on simulation of normal walking patterns [18–21]. Robots for patients with neurological impairment that are purposed to not only strengthen the lower extremities but also exercise the trunk have not yet been sufficiently developed. Regarding the progress monitoring for lower limb rehabilitation in poststroke patients, assessments for gait‐related parameters should be used to determine improvement, which would be better to be assessed automatically during rehabilitation [22].

For this reason, we aimed to develop a robotic system that promotes three‐dimensional (3D) movement of the trunk and gait ability while providing automatic function evaluation and individually optimized prescriptions for stroke patients. We also attempted to discover the shortcomings of the system through user feasibility tests intended to further upgrade the safety concerns of the system and to investigate the potential clinical efficacy for patients with chronic stroke.

## 2. Methods

### 2.1. Participants

Twenty patients with chronic stroke (eight males; mean age, 52.3 ± 11.8 years) who received in‐patient rehabilitation were recruited according to the following criteria: (1) confirmed diagnosis of chronic stroke based on radiologic findings consistent with neurological impairments; (2) poststroke duration of > 12 months; (3) hemiplegia; (4) muscle strength of hip flexor and knee extensor of at least Grade 2 (poor) on Medical Research Council scale on the hemiplegic side; (5) Functional Ambulation Category (FAC) ≥ 2; (6) no change in FAC in screening assessments for 2 months prior to baseline assessment; and (7) ability to follow simple instructions, including how to operate a robotic system (Mini‐Mental State Examination score > 24). The exclusion criteria were as follows: (1) definite musculoskeletal‐origin pain in the lower limbs; (2) severe visuospatial perception problem; and (3) excessive spasticity in the lower limbs (Modified Ashworth Scale score > 2).

This study was approved by the regional Institutional Review Board (The Ethics Committee of the Korea National Institute for Bioethics Policy, IRB No: P01‐202502‐01‐051), and the patients voluntarily consented by written informed consent before participating. This study was registered in the Clinical Research information Service (CRiS) (KCT0010335).

### 2.2. Robotic System

**Figure 1:**
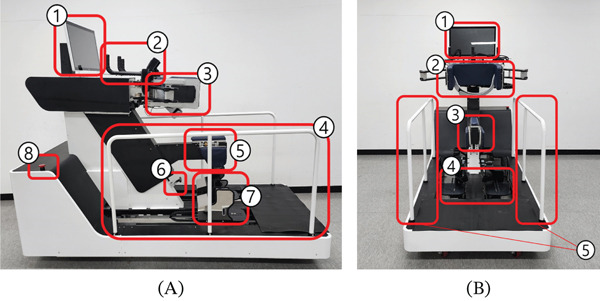
Illustration of the robotic system. Trunk module: (1) user graphic interface, (2) arm supporter, and (3) chest supporter. Upright module: (4) expandable metal pole, (5) saddle, (6) lower extremity module, (7) safety guard, and (8) emergency stop button.

JACOB (LDS Co. Ltd, Daegu, Korea) is a patient‐tailored adaptive trunk–lower limb robotic rehabilitation system and is registered as a medical device (Approval Number 23‐58, 4507). The robot consists of a trunk module, an upright module, and a lower extremity module (Figure 1).

The trunk module consists of arm and chest supporters. The upright module can move up and down while supporting the patient′s body weight and includes a saddle for sitting. The lower extremity module supports the patient′s feet and enables elliptical cycling motion for gait training. The specifications and functions of the components are listed in Table 1.

**Table 1:** System description.

Main parts	Subcomponents	Material and sensors	Function
Trunk module	User graphic interface (1)	Tablet PC	Selecting mode; setting, evaluation, prescription, tutorial, exercise, and result of performance
	Arm supporter (2)	Plastic pole	Fixing the upper limbs for safety
	Chest supporter (3)	Forward bending part with distance sensor rotation part and angle sensor	Holding trunk; sensing forward push, lateral tilt, and rotational movement of the trunk
Upright module	Expandable metal pole (4)	Pneumatic cylinder	Main framework holding the trunk module and saddle and supporting the main load of patients; executing sit‐to‐stand movement with upward movement
	Saddle (5)	Motorized folding in the standing position with weight sensors	Seating area when riding sensing weight during weight bearing and transferring exercise
Lower extremity module	Foot plate (7)	Weight sensors	Holding the feet on standing
	Elliptical cycling module (6)		Gait training
Emergency button	Emergency stop button (8)	Mechanical push button	Immediately stops all device operations in the event of a malfunction; provides hardware‐based emergency stop in compliance with medical device safety requirements

**Figure 2:**
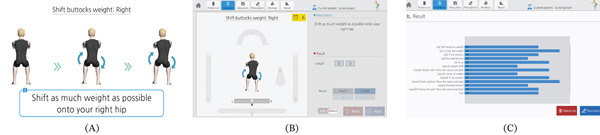
Illustration showing an example of the evaluation and result sheets. (A) Evaluation directive; weight transfer on sitting position. (B) Evaluation result. (C) Result sheet (each evaluation category and its results shown as the score and graph).

A patient with a wheelchair can mount on the system from the backside of the robot and sit on a saddle to assume a seated position. Each patient maintained a comfortable and safe position by placing their chest as close as possible to the chest support and resting their arms on the arm support. The system has a multiple sensing system to measure weight, pressure, angle, and distance parameters for evaluation of patient movements and establishment of treatment protocols. The weight sensors in the saddle measure the amount of support weight loaded on the saddle when the patient transfers their body weight from side to side during the sitting position. The weight sensors in the footplates measure the weight on each footplate in the standing position. Furthermore, the distance, pressure, and angle sensors at the front and on both sides of the chest support enable quantified recognition of the 3D movement of the trunk during forward bending, lateral tilting, or rotation in the sitting or standing position. The upright module supports the entire trunk module, allowing linear motion guidance for the patient to move up and down with the trunk using a pneumatic cylinder. In the standing position, the patient can perform gait training using the elliptical cycling module, which includes foot plates. We adopted a lifting system inside the lower deck to reduce the distance from the floor to the footplate of the robot when the patient mounts the robot. The lower the footplate of the system, the easier it is for the patient to step on it. When initiating gait training, the lifting system lifts the entire system, leaving room for elliptical cycling. Each module can move in only one predetermined direction owing to the design thereof (Figure 2).

The locking system prevents the unwanted movement of each module for each movement of the trunk, and the motor of the locking system is controlled by software. When performing active exercises using only their own muscle strength, patients may use a nonmotor power mode that adds basic mechanical resistance. The setting mode allowed the patients to adjust their sitting position and foot placement to the most comfortable position. This setting was saved automatically to preserve the customized posture for future use.

### 2.3. Clinical and Motor Performance Evaluation Using Kinematic Assessments

Clinical evaluation tools included the Medical Research Council Grade (MRCG) for muscle strength of the hip flexor and knee extensor, modified Ashworth Scale (MAS) for spasticity of the knee flexor on the hemiplegic side, Berg Balance Scale (BBS) for balancing ability, and FAC for gait ability. In this study, cognitive function was assessed using the Korean version of the Mini‐Mental State Examination (MMSE‐K). Permission to use this instrument was granted by Psychological Assessment Resources Inc. (PAR; Lutz, FL, United States).

Kinematic data were obtained using the evaluation mode of the robotic system measuring patients′ capability of weight bearing on the median position, weight transfer on each side of the saddle or foot plate, trunk movement, and gait performance in the sitting, sit to standing, and standing tasks/positions. The trunk movements in the forward, lateral, and rotational directions were assessed within the robot system (Table 2).

**Table 2:** Robotic movements and their corresponding visual guidance on the screen.

Position	Image of movement and graphic features on the screen		
Sitting	Weight transferring to Rt./Lt. 	Forward bending 	Rotation to Rt./Lt. 
	Lateral bending 	Sit‐to‐stand 	
Standing	Weight transferring to Rt./Lt. 	Forward bending 	Rotation to Rt./Lt. 
	Lateral bending 	Gait performance 	

The active range of motion (ROM) of the maximum value for rotation to each side was measured when patient users followed the demonstrative guide on the screen. Weight transfer and trunk lateral bending while sitting or standing were also measured as a percentage score according to the weight or pressure loaded on the affected side compared to that for the unaffected side. The performance results were expressed as a percentage score for the affected versus the unaffected side. For example, when a right‐hemiplegic patient rotates his or her trunk by 30° to the right and 60° to the left, the result of the rotation is 50 points; 100 points means that the affected side could perform as well as the less affected side. Forward bending was measured as the ratio of the maximum distance a patient could bend the trunk forward relative to the device′s provided range of 20 cm.

Gait performance was evaluated by assessing the patient′s ability to follow symmetric gait patterns embedded in the system for 2 min. A typical normal gait pattern model was established based on measurements obtained from 30 healthy adults (80 cycles per person). The gait performance of the patients was expressed as a percentage of the number of gait cycles for 2 min compared to normal gait.

Most performance values were expressed as percentage scores relative to the unaffected side; forward bending was expressed as a percentage of the device‐defined target range. Clinical evaluations and motor performance assessments using the robotic system were conducted twice: before (as a baseline study) and 1 month after the intervention.

### 2.4. Interventions

All participants were instructed by the researchers on how to use the system with written documentation. The researchers demonstrated the system process for evaluation, prescription, exercise, and feedback of patient performance step‐by‐step using the system in front of the participants. The researchers stood by the participants to safeguard the users during the operation and immediately responded to the patients when they were asked for assistance. The robotic intervention was provided as a 30‐min per day, five sessions a week, for 1 month, along with conventional rehabilitation therapy, which was continued as previously described.

**Figure 3:**
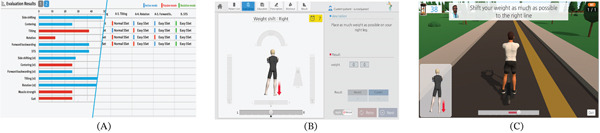
Example of prescription sheet and exercise. (A) Prescription sheet; (B) simple exercise; and (C) game contents.

Based on the kinematic evaluation results, the physician established an individualized rehabilitation exercise program. The exercise sessions were structured as 30‐min interventions, specifically targeting parameters that demonstrated poor performance during the assessment, with a minimum of three repetitions. The parameters of exercise included weight bearing and transfer to each direction, trunk movement to each direction while sitting or standing, sit‐to‐stand movement, and gait training. The physician chose the exercise parameters based on the kinematic data that scored less than 60% of the intact side. The exercise intensity was adjusted according to the score for each parameter (0–30, easy; 30–60, intermediate; and 60–100, hard). At each intensity level, training was conducted with the goal of reaching the maximum value within the designated range. The exercise intensity was intended to be recalibrated at follow‐up assessment (Figure 3).

After completing the intervention, all participants were asked to complete a survey questionnaire with the help of a researcher. The questionnaire included user experiences to evaluate the safety of the device, user comfort, and clinical effects. Each question was responded to as good, moderate, or bad by users (Supporting Information 1).

### 2.5. Statistical Analysis

Statistical analyses were performed using IBM SPSS Statistics software (IBM Corp., Armonk, NY). We used a paired *t*‐test to determine differences before and after the intervention. The significance level was set at 0.05.

## 3. Results

No adverse events, including falls, skin abrasions, muscle strains, or pain exacerbation, were observed during the 1‐month intervention, and all participants completed the program without any device‐related problems. Total 20 participants with chronic stroke had supratentorial lesions, 13 had intracerebral hemorrhage, 7 had infarction, and 9 and 11 had damage to the right and left hemispheres, respectively. The mean period between onset of disease and beginning of study enrollment was 13.5 ± 0.3 months. In the follow‐up evaluation of clinical scales in the MRCG, MAS, BBS, MMSE‐K, and FAC, there were no statistically significant differences between pre‐ and postintervention at 1 month among all participants (Table 3).

**Table 3:** Data of clinical evaluation.

Parameters		Pre	Post 1 month
Muscle strength (MRCs)	Hip flexor	2.95 (0.42)	2.95 (0.42)
	Knee extensor	3.04 (0.33)	3.03 (0.41)
MAS		0.25 (0.37)	0.25 (0.37)
BBS		23.61 (12.79)	23.72 (11.43)
MMSE‐K		26.80 (1.47)	26.80 (1.17)
FAC		2.52 (0.53)	2.57 (0.65)

*Note:* All values presented with number and mean (standard deviation, SD). Pre: pre‐evaluation; post 1 month: post 1 month evaluation.

Abbreviations: BBS, Berg Balance Scale; FAC, Functional Ambulation Category; MAS, modified Ashworth Scale; MMSE‐K, Korean version of the Mini‐Mental State Examination; MRC, Medical Research Council Scale.

In contrast, several kinematic parameters demonstrated significant improvements after 1 month of intervention (Table 4). In the sitting position, lateral bending increased from 42.55 ± 25.52 to 46.25 ± 26.50, and forward bending increased from 12.9 ± 5.33 to 23.23 ± 7.13 (*p* < 0.05). During the sit‐to‐stand task, forward bending significantly improved from 15.0 ± 3.52 to 26.1 ± 5.19 (*p* < 0.05). In the standing position, weight transfer increased from 45.15 ± 12.59 to 57.8 ± 9.47, forward bending from 12.85 ± 5.97 to 23.8 ± 12.80, and lateral bending from 44.65 ± 26.83 to 53.85 ± 25.14 (all *p* < 0.05). However, no significant changes were observed in sitting weight bearing (72.0 ± 16.36 vs. 73.05 ± 15.44), sitting weight transfer, trunk rotation ROM in sitting or standing, standing weight bearing, or gait performance (39.4 ± 21.09 vs. 40.25 ± 17.49) (Table 4).

**Table 4:** Comparison between baseline and post 1 month intervention of kinematic assessment.

Position	Movement	Pre	Post 1 month
Sitting	Weight bearing	72 (16.36)	73.05 (15.44)
	Weight transfer	47.2 (12.98)	48 (12.57)
	Lateral bending	42.55 (25.52)	46.25 (26.50) ^∗^
	Rotation	58.25 (18.53)	59.95 (17.26)
	Forward bending	12.9 (5.33)	23.23 (7.13) ^∗^
Sit‐to‐stand	Forward bending	15 (3.52)	26.1 (5.19) ^∗^
Standing	Weight bearing	71 (10.87)	69.5 (11.09)
	Weight transfer	45.15 (12.59)	57.8 (9.47) ^∗^
	Forward bending	12.85 (5.97)	23.8 (12.80) ^∗^
	Rotation	53.95 (20.39)	55 (21.20)
	Lateral bending	44.65 (26.83)	53.85 (25.14) ^∗^
	Gait performance	39.4 (21.09)	40.25 (17.49)

*Note:* All values presented with number and mean (standard deviation, SD). Pre: pre‐evaluation; post 1 month: post 1 month evaluation.

^∗^ *p* < 0.05.

The results of the user feasibility survey showed that 16 out of 20 patients (80%) were satisfied with the easy and safe and easy access to the system, sitting activity, sit‐to‐stand activity, standing activity, and gait training activity. Moderate and negative marks were recorded across all five categories (Access, On sitting, Sit‐to‐stand, On standing, and Gait trainer), totaling 13 out of 100 responses (13%), all of which were related to the safety‐blocking system, which is designed to prevent mechanical collision—either among the device′s components or between the device and the patient′s body—given the device cannot be fully individualized to each patient′s anthropometric characteristics (Table 5).

**Table 5:** The result of user feasibility test.

Categories	Good	Moderate	Bad	Total (*N* = 20)
Access	90	10	0	
On sitting	85	10	5	
Sit‐to‐stand	85	15	0	
On standing	80	10	10	
Gait trainer	95	5	0	

*Note:* All values presented with percentage of the responses.

Accordingly, the locking system was updated, including replacement of manual components with motor‐driven components, for user convenience. These improvements required additional firmware and software updates.

## 4. Discussion

In this study, we investigated the effects of a trunk–lower limb robotic system on muscle strength, balance, and functional status during a 1‐month training period in chronic stroke patients. The main finding of this pilot investigation is that trunk‐focused robotic training improved several kinematic parameters related to trunk control, whereas conventional clinical scales and gait ability remained unchanged over the short intervention period. In the follow‐up evaluation of the clinical evaluation scores of MRCG, MAS, BBS, MMSE‐K, and FAC, no significant improvements were observed among the total participants. In contrast, meaningful improvements were found in the assessments from the robotic system; lateral and forward bending trunk ROM while sitting, forward bending during sit‐to‐stand task, weight transfer and trunk ROM of lateral bending, and forward bending while standing. These findings suggest that the intervention preferentially enhanced segmental trunk control rather than global functional recovery as measured by conventional clinical scales.

Trunk control and balance stability are core components of human locomotive function. Proper trunk control enables sit‐to‐stand transitions and is associated with stable walking and a reduced risk of falling [13–15]. Therefore, trunk balance training constitutes an important aspect of the rehabilitation process for various neurologic diseases. Trunk control is also recognized as an important early indicator of activities of daily living after a stroke [13]. Robust evidence demonstrates that training in trunk control and core stability can enhance sitting and standing balance, mobility, trunk stability, and neuromuscular coordination. Moreover, improving trunk regulation may lead to increased proprioception, dynamic balance, walking speed, and symmetrical trunk movements during gait in stroke survivors [23–26]. From a biomechanical perspective, trunk control plays a critical role in providing proximal stability for distal limb movement [27, 28]. Adequate trunk stability facilitates anticipatory postural adjustments and effective weight transfer [29–31], both of which are essential prerequisites for coordinated lower limb movement during functional activities such as sit‐to‐stand transitions and gait initiation. Therefore, robotic training that specifically targets trunk movement may contribute to improving foundational postural control mechanisms in stroke rehabilitation.

Additionally, we found no significant changes in weight bearing, transfer, trunk rotation ROM, and gait speed parameters obtained from the robotic system. The absence of improvement in gait‐related outcomes suggests that short‐term trunk‐focused robotic training may not be sufficient to induce measurable improvements in complex locomotor functions in chronic stroke patients. The results indicate that the rehabilitation robot system could improve several functional performances, especially trunk movements, but not the rotation component, whereas the gait speed and scores of clinically used evaluation tools and other kinematically achieved data did not change. One possible explanation is that improvements in segmental trunk control may not immediately translate into functional locomotor recovery without sufficient training duration or task‐specific gait practice. Chronic stroke patients often require intensive and prolonged rehabilitation to achieve meaningful functional changes. Therefore, a longer intervention period or integration with task‐specific gait training protocols may be necessary to translate trunk improvements into clinically meaningful gains in walking ability.

Compared with existing gait‐centered robotic systems, our findings highlight the potential value of targeting trunk control as a foundational component of poststroke rehabilitation. Many currently available robotic systems primarily focus on reproducing lower limb gait patterns [6, 32, 33], whereas deficits in proximal trunk stability are often overlooked despite their critical role in maintaining balance and coordinating limb movements. Rather than solely simulating gait patterns, trunk‐focused interventions may address proximal stability deficits that underlie impaired mobility. In this context, the present robotic system was designed to promote multidirectional trunk movements during both sitting and standing conditions, which may facilitate more active engagement of postural control mechanisms during rehabilitation. Regarding engineering features, rehabilitation robots for gait training are generally classified into end‐effector and exoskeleton types. End‐effector systems typically guide lower limb movement through footplates, whereas exoskeleton systems provide powered assistance through wearable joint actuators [23–26, 34–37]. In contrast to these conventional designs that primarily focus on lower limb movement, the present system was designed to facilitate active multidirectional trunk control during both sitting and standing positions. The upright support module also adopts a piggyback‐like suspension structure, allowing patients who have difficulty maintaining independent sitting or standing to be comfortably supported during training. This design enables targeted training of trunk movements, including forward bending, lateral tilting, and rotation, which are essential for postural stability and functional mobility in stroke rehabilitation.

As results, the selective improvement observed in lateral and forward bending, but not in trunk rotation, may indicate that sagittal and frontal plane movements are more responsive to short‐term repetitive robotic training than transverse plane control. Rotational trunk movements typically require more complex coordination between trunk segments and pelvic control, which may explain the relatively limited responsiveness of this parameter within a short training period. This observation may help refine future training protocols to better incorporate rotational components. In this study, robotic‐derived kinematic parameters captured improvements that were not detected by conventional clinical scales. This finding highlights the potential advantage of objective motion‐based metrics in detecting subtle motor changes during rehabilitation. Conventional clinical scales such as BBS or FAC are ordinal measures and may have limited sensitivity to detect small improvements in segmental motor performance. In contrast, robotic systems equipped with motion sensors can provide continuous quantitative data on movement performance, enabling more sensitive monitoring of rehabilitation progress. The improvement in specific trunk kinematic parameters, despite unchanged global clinical scales, suggests that robotic‐derived metrics may complement traditional functional assessments in evaluating rehabilitation outcomes.

Despite the system′s emphasis on active and rhythmic weight transfer, gait performance did not significantly improve. This finding suggests that improvements in isolated trunk control do not necessarily translate directly into complex locomotor function within a short time frame. Longer intervention duration or integration with task‐specific gait training may be necessary to achieve functional transfer. Therefore, combining trunk‐focused robotic training with more intensive gait‐specific interventions may enhance the functional transfer of trunk improvements to real‐world walking performance.

As we sought to investigate the clinical safety and potential usefulness of our system in advance before conducting a control study, we recruited chronic patients who had not shown significant change in gait ability prior to study enrollment. This design choice reduces the likelihood that the observed improvements were due to spontaneous recovery. A short‐term intervention period of 1 month was selected to explore whether measurable changes in kinematic parameters could be detected after robotic training. Although improvements in several trunk‐related parameters were observed, the short duration of intervention should be considered when interpreting the absence of improvement in gait‐related outcomes.

In this clinical trial, we investigated the features and requirements of robotic systems used to provide treatments in the clinical field as perceived by medical personnel and primary users, including patients, therapists, and physicians. In the feasibility assessment, most responses were rated as good, including those related to overall satisfaction and ease of system use. The feedback from users implies that the proposed system is safe and can be operated easily. Users were dissatisfied with the manipulation of the safety‐blocking system and expressed a need to improve the system in this regard. We upgraded the manually operated locking system to a motor‐driven system, which enabled more convenient operation and automatic adjustment of the software settings. Thanks to valuable information from the user feasibility test, we developed the final version of the commercialized system. We plan to conduct a clinical trial on chronic stroke patients with a controlled study design and long‐term therapy sessions.

## 5. Limitations

There are certain limitations to this pilot study. First, this study was not randomized controlled trial and did not include control group. The absence of a control group limits the interpretability of the results, especially regarding causality. Second, only individuals in the chronic phase poststroke, with a small sample size, were enrolled. The small sample size may not provide adequate representation of stroke heterogeneity, including lesion location, severity and functional impairment. Thirdly, the feasibility and satisfaction surveys, while positive, were solely on self‐report and lack standardization or validation. Future studies should improve methodological rigor by incorporating standardized usability instruments, such as the System Usability Scale, along with objective usability measures.

## 6. Conclusion

In this short‐term pilot study of patients with chronic stroke, the robotic system was associated with improvements in selected trunk‐related kinematic parameters, whereas gait ability and conventional clinical scales did not significantly change. The system was generally safe and feasible to use, and user feedback informed further refinement of the device. Controlled studies with longer intervention periods are needed to determine whether improvements in trunk performance can translate into functional gait recovery.

## Funding

This study was supported by a grant from the Translational R&D Program on Smart Rehabilitation Exercises (TRSRE‐EQ02A), National Rehabilitation Center, and Ministry of Health and Welfare, Republic of Korea.

## Ethics Statement

This study was conducted in accordance with the Declaration of Helsinki and approved by the regional Institutional Review Board (IRB No.: P01‐202502‐01‐051).

## Consent

All the patients voluntarily consented by written informed consent before participating.

## Conflicts of Interest

The authors declare no conflicts of interest.

## Supporting Information

Additional supporting information can be found online in the Supporting Information section.

## Supporting information


**Supporting Information 1** (Questionnaire). User feasibility test questionnaire administered to evaluate participants′ experience with the device, covering access to the system, sitting position, sit‐to‐stand, standing position, and gait training.

**Supporting Information 2** Figure S1: Examples of weight‐shifting training tasks used in the rehabilitation program.

**Supporting Information 3** Figure S2: Examples of pelvic weight‐shifting training tasks used in the rehabilitation program.

**Supporting Information 4** Figure S3: Examples of trunk control training tasks used in the rehabilitation program.

**Supporting Information 5** Figure S4: Rehabilitation training in the seated position using the rehabilitation device.

**Supporting Information 6** Figure S5: Sit‐to‐stand training using the rehabilitation device.

**Supporting Information 7** Figure S6: Gait and stepping training using the rehabilitation device.

## Data Availability

The data that support the findings of this study are available on request from the corresponding author. The data are not publicly available due to privacy or ethical restrictions.
