# Supplementary material for: Development and Upgrade of a Robot That Enables Three‐Dimensional Trunk Motion and Lower Limb Exercise for Stroke Patients: A Pilot Clinical Investigation Including a User Feasibility Test
Source: Biomed Res Int. 2026 Aug 5;2026:5515943. doi: 10.1155/bmri/5515943 (PMC13439226; doi:10.1155/bmri/5515943)
Supplement: Supplementary file 1 [file BMRI-2026-5515943-s001.docx]

**Supplementary Materials**

User feasibility test questionnaire

A. Access to the system

1. Do you need a step to hop on?

2. Is the height of the base platform okay?

3. Is the safety guard helpful?

4. Is the saddle and foot plate in a good position to sit on?

5. Is it easy to adjust the width of the chest supporter?

6. How many helpers do you need to use the system?

B. On sitting position

1. Is the height of the saddle optimal?

2. Is it easy to operate the safety block?

3. How is the width between both foot plates?

4. Is the initial position of the chest supporter good for you?

5. How is the design of the forearm supporter? Is it good to hold a hemiplegic hand?

6. Is it easy to manipulate the forward bending locking system?

7. Is the distance of forward bending good?

8. Is it easy to manipulate the rotation locking system?

9. Is it optimal to rotate? Do you need too much effort to rotate?

10. Is the maximal angle of rotation good?

C. Sit-to-stand

1. Is it easy to adjust the position of the foot plate?

2. How is the angle of inclination of the upright? Is it good?

3. How is the strength of the gas cylinder of the upright? Is it good?

4. How is the distance between the foot plate and the upright? Is it comfortable?

D. On standing position

1. Same as questions on sitting position

2. Is there any interference between the saddle and your thigh?

E. Gait trainer

1. Is the position of the foot plate good?

2. Is it easy to operate the locking system?

Supplementary Figure 1. Examples of weight-shifting training tasks used in the rehabilitation program

Supplementary Figure 2. Examples of pelvic weight-shifting training tasks used in the rehabilitation program

Supplementary Figure 3. Examples of trunk control training tasks used in the rehabilitation program

Supplementary Figure 4. Rehabilitation training in the seated position using the rehabilitation device

Supplementary Figure 5. Sit-to-stand training using the rehabilitation device

Supplementary Figure 6. Gait and stepping training using the rehabilitation device
